# *TCF21* hypermethylation regulates renal tumor cell clonogenic proliferation and migration

**DOI:** 10.1002/1878-0261.12149

**Published:** 2017-12-14

**Authors:** Saskia L. Gooskens, Timothy D. Klasson, Hendrik Gremmels, Ive Logister, Robert Pieters, Elizabeth J. Perlman, Rachel H. Giles, Mary M. van den Heuvel‐Eibrink

**Affiliations:** ^1^ Princess Máxima Center for Pediatric Oncology Utrecht The Netherlands; ^2^ Department of Pediatric Hematology and Oncology Erasmus MC – Sophia Children's Hospital Rotterdam The Netherlands; ^3^ Department of Nephrology and Hypertension University Medical Center Utrecht University of Utrecht The Netherlands; ^4^ Department of Pathology Ann and Robert H. Lurie Children's Hospital of Chicago Northwestern University's Feinberg School of Medicine and Robert H. Lurie Cancer Center IL USA

**Keywords:** clear cell sarcoma of the kidney, epithelial‐to‐mesenchymal transition, methylation, renal cell carcinoma, TCF21, tumor suppressor

## Abstract

We recently identified hypermethylation at the gene promoter of transcription factor 21 (*TCF21*) in clear cell sarcoma of the kidney (CCSK), a rare pediatric renal tumor. *TCF21* is a transcription factor involved in tubular epithelial development of the kidney and is a candidate tumor suppressor. As there are no *in vitro* models of CCSK, we employed a well‐established clear cell renal cell carcinoma (ccRCC) cell line, 786‐O, which also manifests high methylation at the *TCF21* promoter, with consequent low *TCF21* expression. The tumor suppressor function of *TCF21* has not been functionally addressed in ccRCC cells; we aimed to explore the functional potential of *TCF21* expression in ccRCC cells *in vitro*. 786‐O clones stably transfected with either pBABE‐TCF21‐HA construct or pBABE vector alone were functionally analyzed. We found that ectopic expression of *TCF21* in 786‐O cells results in a trend toward decreased cell proliferation (not significant) and significantly decreased migration compared with mock‐transfected 786‐O cells. Although the number of colonies established in colony formation assays was not different between 786‐O clones, colony size was significantly reduced in 786‐O cells expressing *TCF21*. To investigate whether the changes in migration were due to epithelial‐to‐mesenchymal transition changes, we interrogated the expression of selected epithelial and mesenchymal markers. Although we observed upregulation of mRNA and protein levels of epithelial marker E‐cadherin in clones overexpressing *TCF21*, this did not result in surface expression of E‐cadherin as measured by fluorescence‐activated cell sorting and immunofluorescence. Furthermore, mRNA expression of the mesenchymal markers vimentin (*VIM*) and *SNAI1* was not significantly decreased in *TCF21*‐expressing 786‐O cells, while protein levels of *VIM* were markedly decreased. We conclude that re‐expression of *TCF21* in renal cancer cells that have silenced their endogenous *TCF21* locus through hypermethylation results in reduced clonogenic proliferation, reduced migration, and reduced mesenchymal‐like characteristics, suggesting a tumor suppressor function for transcription factor 21.

**Abbreviations**
CCSK: clear cell sarcoma of the kidney
EMT: epithelial‐to‐mesenchymal transition
FACS: fluorescence‐activated cell sorting
MET: mesenchymal‐to‐epithelial transition
MFI: median fluorescence intensity
RCC: renal cell carcinoma
TARGET: therapeutically applicable research to generate effective treatments
TCF21: transcription factor 21

## 1 Introduction

Clear cell sarcoma of the kidney (CCSK) is an uncommon pediatric renal tumor that comprises between 2% and 5% of all primary renal tumors in children (Furtwangler *et al*., 2013; Gooskens *et al*., 2012; Mullen *et al*., 2014). This tumor is observed most often in children between 2 and 4 years of age and is characterized by a high malignant potential (Argani *et al*., 2000; Gooskens *et al*., 2012). Apart from internal tandem duplications within exon 16 of *BCOR* (reported in the majority of CCSKs) and translocation t(10;17)(q22;p13) leading to a fusion of *YWHAE* and *NUTM2B/E* (reported in about 10% of CCSKs), the genome of CCSK seems to be rather stable (Astolfi *et al*., 2015; Gooskens *et al*., 2015; Karlsson *et al*., 2015; Roy *et al*., 2015; Ueno‐Yokohata *et al*., 2015). This paucity of large genomic imbalances and the few detected somatic mutations in CCSK prompted the investigation of the epigenome of CCSKs. Our study as part of the NCI‐initiated therapeutically applicable research to generate effective treatment project (TARGET) identified hypermethylation of the 5′ region of transcription factor 21 (*TCF21*) in all studied CCSK samples, except for samples harboring the *YWHAE‐NUTM2* fusion transcript (Gooskens *et al*., 2015). This hypermethylation was negatively correlated with *TCF21* expression. Other tested pediatric renal tumor samples and normal kidney samples showed significantly lower *TCF21* methylation levels.

*TCF21* (also referred to as *Pod‐1, capsulin*, and *epicardin*) encodes a basic helix‐loop‐helix transcription factor that binds DNA and regulates cell differentiation and cell‐fate specification during development (Hidai *et al*., 1998). It is expressed in embryonic mesenchymal cells surrounding areas of epithelial development in the kidney, heart, lung, and gastrointestinal tract (Hidai *et al*., 1998; Lu *et al*., 1998; Quaggin *et al*., 1998). *TCF21* expression rapidly decreases in postnatal tissues with the exception of a subset of interstitial cells in organs including the kidney, heart, lung, and spleen (Plotkin and Mudunuri, 2008). Antisense inhibition of *TCF21* has been reported to disrupt epithelial differentiation and branching morphogenesis of the epithelium in murine embryonic kidney, suggesting a role for *TCF21* in epithelial–mesenchymal interactions (Quaggin *et al*., 1999). Gene deletion studies in chimeric mice have shown that loss of *TCF21* in the kidney results in decreased glomerulogenesis and tubulogenesis (Cui *et al*., 2003). Of note, suppression of *TCF21* expression by siRNA within a mouse kidney progenitor cell line that endogenously expresses *TCF21* resulted in increased cell proliferation and migration, as well as reduced expression of smooth muscle genes and myofibroblast secreted proteins (Plotkin and Mudunuri, 2008).

Currently, no CCSK cell lines or models are available to functionally verify the role of *TCF21* hypermethylation in this renal tumor type. Therefore, we searched for an alternative model. A literature search revealed that *TCF21* hypermethylation is also present in clear cell renal cell carcinomas (ccRCC): renal tumors with another biology and phenotype, which most often occur in adults (Costa *et al*., 2011; Ye *et al*., 2012). Previously performed clinical studies on the prognostic impact of *TCF21* expression in ccRCC tissue revealed that *TCF21* expression levels significantly correlated with Fuhrman nuclear grade and cancer‐specific survival of ccRCC patients (Ye *et al*., 2012). *TCF21* methylation levels in urine samples were significantly correlated with tumor size, Fuhrman grade, and clinical stage (Xin *et al*., 2016). However, no studies have functionally addressed the tumor suppressor activity of *TCF21* in renal cancer cells. Therefore, the aim of this study was to explore the functional potential of *TCF21* expression in the tumorigenesis of ccRCC *in vitro*.

## 2 Methods

### 2.1 Cancer cell lines

We used the human ccRCC 786‐O cell line (ATCC CRL‐1932). 786‐O cells are reported to manifest high methylation and low expression of *TCF21* (Costa *et al*., 2011). Cells were cultured in Gibco RPMI Medium 1640 (1X) + GlutaMAX™‐I (Invitrogen, Carlsbad, CA, USA), supplemented with 10% fetal bovine serum (Invitrogen), penicillin (50 IU·mL^−1^), and streptomycin sulfate (50 μg·mL^−1^; Invitrogen).

### 2.2 Vector construction and transfection

The coding sequence of *TCF21* (including HA‐tag) was cloned out of a pCS2+‐TCF21 construct [kindly provided by Prof. Christopher Plass and Khalifa Arab, Division of Epigenomics and Cancer Risk Factors, German Cancer Research Center (DKFZ), Heidelberg, Germany], amplified, and recloned into a pBABE‐puro vector. Plasmid DNAs were sequence‐confirmed. Twenty‐five micrograms of pBABE‐TCF21‐HA or pBABE‐puro vector alone was transfected into cells of the 786‐O cell line using electroporation. Electroporation was performed in a 4‐mm gap cuvette (#165‐2088; Bio‐Rad Laboratories, Hercules, CA, USA) using a Gene Pulser (Bio‐Rad, München, Germany) with electric parameters 24 kV with 1000 uF capacitance; a single exponential decay pulse was applied. Selection medium containing puromycin was added to the cells after 48 h of recovery, and colonies grew after 2 weeks of culture. Eight colonies were selected for functional assays: four from pBABE‐puro mock‐transfected 786‐O cells (N1F4, B5F1, N1G4, and B6D10) and four expressing HA‐tagged exogenous *TCF21* (2B12, 5D2, 9D12, and 9H9). Unless the clones are specifically named, data from ‘pBABE‐mock’ or ‘pBABE‐TCF21’ contain pooled data from all four clones.

### 2.3 Western blotting

Cells were lysed on ice in RIPA buffer and normalized to 40 μg of protein per sample. Lysates were loaded and fractionated by SDS/PAGE (14% gel) under protein‐reducing conditions and immunoblotted on poly(vinylidene difluoride) (PVDF) membranes. β‐Actin or β‐tubulin was used as loading control. After blotting, the PVDF membranes were blocked in 5% dried skim milk in TBS with 0.5% Tween. Primary antibodies used were monoclonal mouse anti‐HA (supernatant from hybridoma clone 12CA5) at a dilution of 1 : 3, rabbit polyclonal anti‐TCF21 (32981, 1 : 10 000; Abcam, Cambridge, UK), rabbit polyclonal anti‐E‐cadherin (15148, 1 : 500; Abcam), rabbit anti‐VIM (VIM; R28; 3932, 1 : 200; Cell Signaling, Danvers, MA, USA) with loading controls being anti‐actin (mouse anti‐β‐actin AC‐15, Sigma A5441, 1∶15 000) or anti‐β‐tubulin (rabbit anti‐β‐tubulin Cat. 2128, 1 : 1000; Cell Signaling). ECL reagent (Amersham Biosciences) was used for detection with ImageQuant LAS 4000 (GE Healthcare, Hoevelaken, the Netherlands).

### 2.4 Proliferation assay

A total of 2 × 10^4^ 786‐O pBABE‐mock cells (four different clones) and 786‐O pBABE‐TCF21 cells (four different clones) were plated in 6‐well plates in two separate experiments in triplicate in selection medium and were counted after 2, 4, and 5 days. Cells were counted in duplicate with the Bio‐Rad TC20 Automated Cell Counter (Hercules, CA, USA) and averaged.

### 2.5 Colony formation assay

A total of 200 786‐O pBABE‐mock cells (four different clones) and 786‐O pBABE‐TCF21 cells (four different clones) were plated in 6‐well plates on five different occasions in triplicate in selection medium until 786‐O pBABE‐mock cells formed sufficiently large colonies (> 50 cells per colony, 10–14 days). Cells were then washed with PBS and fixed and stained using a mixture of 6.0% glutaraldehyde and 0.5% crystal violet for 30 min at room temperature, before being washed in water. Wells were individually photographed and analyzed by the Colony Counter plug‐in for Image J software. Numbers of colonies and colony area were calculated in Image J.

### 2.6 Migration assays

Boyden chambers (Transwell Permeable Supports; Cole‐Parmer; BergmanLabora AB, Danderyd, Sweden) were used to evaluate the migratory capacity of 786‐O pBABE‐mock cells (N1G4 and B6D10 clones) and 786‐O pBABE‐TCF21 cells (9D12 and 5D2 clones). 3 × 10^4^ cells were seeded in the upper section of a Boyden chamber containing serum‐free medium. The lower section, separated from the upper one by a membrane with 8‐μm pores, contained medium with 10% serum. Cells were grown at 37 °C for 16 h, fixed in methanol, and stained with DAPI (1 : 2000). Unmigrated cells on top of the membrane were removed with a cotton tip. The number of migrated cells on the bottom of the membrane was evaluated by counting cells in 10 different views per membrane using microscopy at 10× magnification. The experiments were performed in duplicate and were repeated two times (first time N1G4/B6D10 versus 9D12/5D2, second time N1G4 versus 5D2).

For the real‐time migration assay, in each experiment, a modified Boyden chamber setup was used, with 10% serum in the lower well as chemoattractant. Migration was recorded in real‐time at 5‐min intervals over 24 h using the xCELLigence Real‐Time Cell Analysis system (Acea Biosciences, San Diego, CA, USA), which quantifies cell numbers in the lower compartment by electrical impedance as reported by Gremmels *et al*. (2014). Rate of migration was quantified by calculating a series of moving slopes over 12 interval periods and taking the maximum slope in each migration curve. The moving slope takes the slope over small sections of the S‐curve, 12 intervals of 5 min. So one takes the slope over the 0‐ to 60‐min interval, 5‐ to 65‐min, 10‐ to 70‐min, 15‐ to 75‐min interval, and so on until the last point. Of the values, the highest value is the maximum slope for a given curve, which we used for analysis.

Within each experiment, four technical replicates per clone were recorded and the experiment was repeated on three separate days. Data were analyzed using a hierarchical linear mixed model using experimental day as a random variable.

### 2.7 Zebrafish injections

An incross of *vhl*+/− zebrafish embryos (van Rooijen *et al*., 2009) carrying a transgene for GFP‐labeled *cadherin‐17* (kidney‐specific) at the 1–2 cell stage were injected with 1–2 nL of mRNA (concentration 50 ng·μL^−1^) transcribed from the linearized pCS2+‐TCF21 plasmid (SP6 mMessage mMachine kit; Ambion, Oudeschoot, the Netherlands) in pure water with 0.05% Phenol Red using a nanoject2000 microinjector (World Precision Instruments, Sarasota, FL, USA). Pronephros volume (determined by GFP fluorescence) was measured at 5.75 days postfertilization and imaged with an LSM700 confocal microscope (Zeiss, Oberkochen, Germany). All embryos were genotyped after imaging as previously described (van Rooijen *et al*., 2009), and only confirmed *vhl*−/− embryos were analyzed. All zebrafish experiments were approved by the Animal Care Committee of the University Medical Center Utrecht, the Netherlands.

### 2.8 Quantitative RT‐PCR

Total RNA was extracted and purified using the RNeasy Mini Kit (74106; QIAGEN, Hilden, Germany). cDNA was synthesized from 1 μg of RNA by the iScript cDNA Synthesis Kit (170‐8891; Bio‐Rad) according to the supplier's protocol. A cDNA concentration of 5 ng·μL^−1^ was used for the quantitative RT‐PCRs; samples were run with iQ SYBR Green Supermix (170‐8880; Bio‐Rad) and CFX96 Touch Real‐Time PCR Detection System (Bio‐Rad); 95 °C for 3 min, followed by 40 cycles of 10 s of 95 °C, 10 s of 50–65 °C and 20 s of 72 °C, then 10 s at 95 °C followed by a melt of the product from 65 to 95 °C. Values were normalized to the housekeeping gene *GAPDH*. All experiments were performed in triplicate on two different dates, from independent mRNA harvests. Relative expression ratios of target genes were calculated using the comparative cycle threshold method (Vandesompele *et al*., 2002). Primer sequences are provided in Table S1.

### 2.9 Fluorescence‐activated cell sorting (FACS)

We trypsinized cells from each clone and stained unfixed live cells with anti‐E‐cadherin (Cat # 563571; BD Biosciences, Vianen, the Netherlands) and anti‐CD24 (Cat # 562789; BD Biosciences). Ten thousand events were acquired in a FACSCanto II flow cytometer (BD Biosciences). Representative samples were plotted as histograms; median fluorescence intensity (MFI) of each channel was used for quantification.

### 2.10 Immunofluorescence

Coverslips were seeded with cells at low (50% confluent) or high density (confluent) and cultured for 24 h with medium containing serum, followed by 40 h with serum‐free medium. Cells were rinsed with PBS and fixed with 4% PFA for 15 min at room temperature, followed by permeabilization with 1% bovine serum albumin/0.1% Triton X‐100 in PBS. Cells were stained with rabbit anti‐E‐cadherin (15148, 1 : 500; Abcam) for 4 h at room temperature. Secondary antibody Alexa Fluor‐488 anti‐rabbit (1 : 500; Invitrogen) was incubated at room temperature for 1 h. Z‐stacks for 10 cells per condition were generated using a Zeiss LSM700 confocal microscope and photographed using Zeiss Zen black edition software (Jena, Germany). Maximum projected images of representative cells are shown.

### 2.11 Statistical analysis

**Figure 1:**
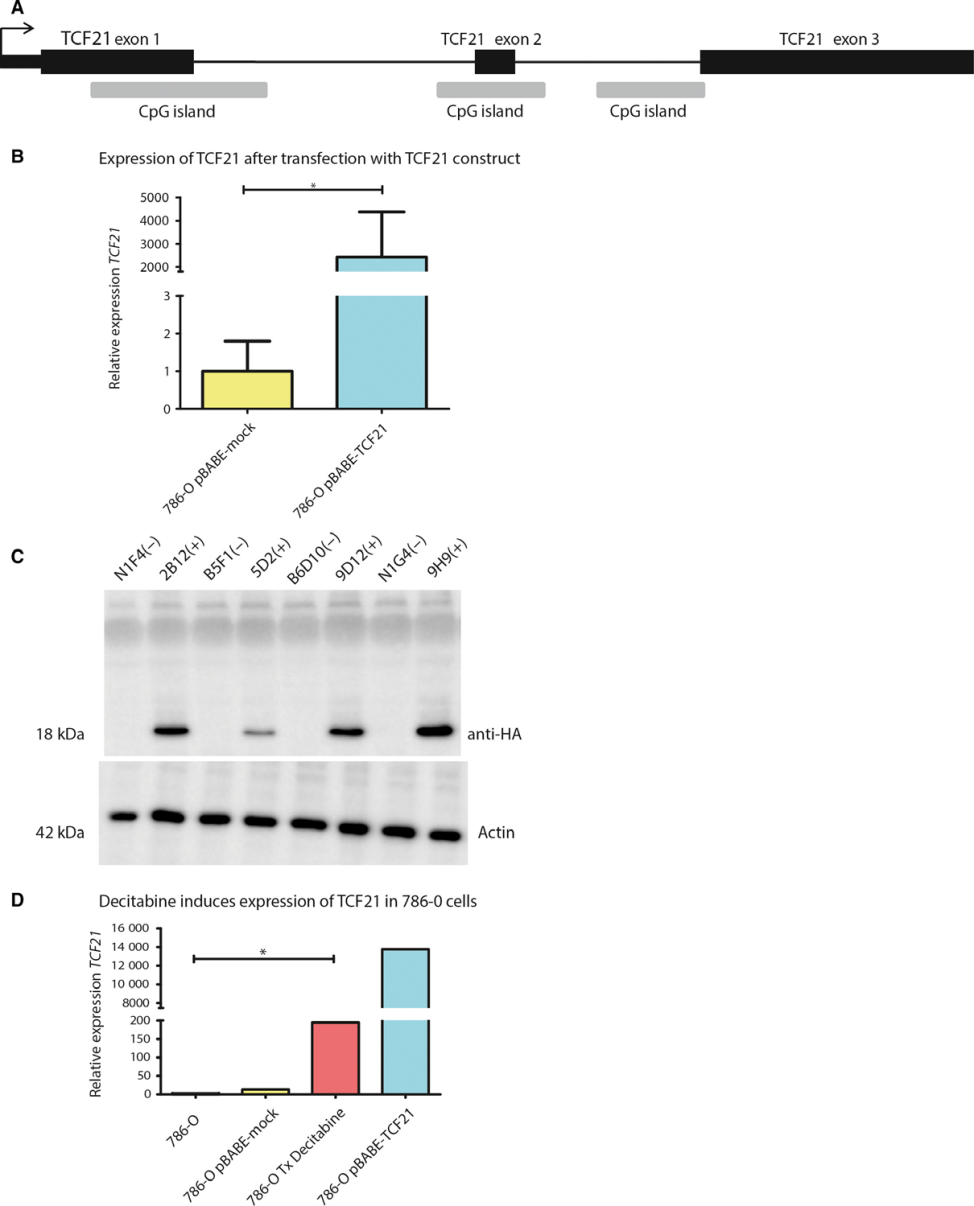
*TCF21* methylation, expression, and reconstitution. (A) Schematic of the *TCF21* gene, showing relative position of the CpG islands to the first three exons. (B) *TCF21* mRNA expression levels in 786‐pBABE‐mock and 786‐O pBABE‐TCF21 cells, as measured by qPCR. Data are presented as mean ± SEM. *N* = 2 independent experiments, each performed in triplicate. (C) Western blots of eight clones of 786‐O that were used for this study; four clones ‘(−)’ were mock‐transfected with pBABE‐puro (N1F4, B5F1, B6D10, and N1G4) and four ‘(+)’ were transfected with HA‐tagged pBABE‐TCF21 (2B12, 5D2, 9D12, and 9H9). Upper blot was immunostained with anti‐HA to detect exogenous TCF21 (18 kDa), while lower blot is loading control stained with anti‐actin (42 kDa). *N* = 2 independent experiments, representative result shown. (D) Treating 786‐O cells with 3 μm decitabine increases the expression of *TCF21* mRNA, as measured by qPCR. *N* = 1, performed in duplicate. *P < 0.05.

Statistical analyses were performed by the two‐tailed Student's *t*‐test using GraphPad, except for statistical analysis of the real‐time migration assay (see paragraph 2.6) and analysis of qPCR *TCF21* values as shown in Fig. 1D, which were analyzed using one‐way ANOVA. Differences were considered statistically significant at *P* < 0.05 (*) and *P* < 0.01 (**).

## 3 Results

### 3.1 786‐O ccRCC cells have low endogenous levels of *TCF21*

We first asked whether *TCF21* methylation (Fig. 1A) could impact ccRCC cell proliferation. To this end, we generated isogenic stable cell lines for *in vitro* comparison of cell proliferation in 786‐O cells transfected with an empty pBABE vector (‘pBABE‐mock’ clones N1F4, B5F1, B6D10, N1G4) and 786‐O cells stably expressing pBABE‐TCF21 (clones 2B12, 5D2, 9D12, 9H9). *TCF21* mRNA and protein expression is very low to undetectable in control 786‐O cells by qPCR or by western blot (Fig. S1). Overexpression of *TCF21* was examined by qPCR (Fig. 1B) and by western blot (Fig. 1C, Fig. S1). Treatment of 786‐O cells with 3 μm of the demethylating agent decitabine (5‐aza‐2′‐deoxycytidine) for 96 h increased *TCF21* mRNA levels (Fig. 1D), supporting data from Ye *et al*. (2012) that the endogenous low level of *TCF21* in 786‐0 cells can be at least partly explained by methylation of the *TCF21* promoter.

### 3.2 *TCF21* ectopic expression suppresses clonogenic cell proliferation

**Figure 2:**
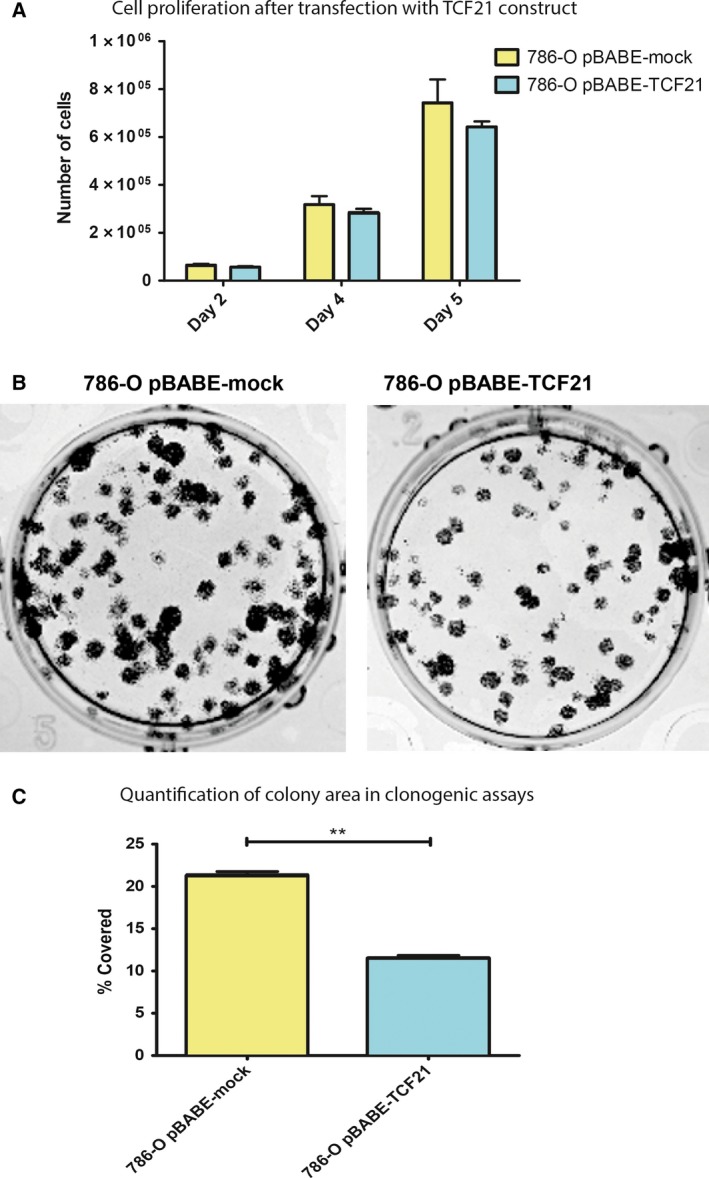
*TCF21* expression reduces clonogenic cell proliferation. (A) Pooled data from all four clones either pBABE‐mock or pBABE‐TCF21 for cell counts on day 2, 4, or 5 showing an insignificant trend toward lower proliferation in 786‐O pBABE‐TCF21 cells. Data are presented as mean ± SEM. *N* = 2 independent experiments, each performed in triplicate. (B) Representative photographs of wells from clonogenic assays. (C) Quantification of area covered by colonies in clonogenic assays. Data are presented as mean ± SEM. *N* = 5 independent experiments, each performed in triplicate. **P < 0.01.

To investigate whether *TCF21* expression results in a reduction in cell proliferation in the context of the ccRCC cell line 786‐O, we performed standard growth curves on the four clones that were pBABE‐mock‐transfected and compared them to standard growth curves from the four clones expressing *TCF21* after 2, 4, and 5 days. Although the difference in proliferation was not significant at any of the chosen time points, we observed a mild trend toward decreased proliferation in the pBABE‐TCF21 clones (Fig. 2A). We therefore suggest that *TCF21* expression moderately suppressed proliferation of this 786‐O cancer cell line and that the hypothesis that proliferation is suppressed by *TCF21* warranted further investigation.

We next assessed the ability of our clones to generate colonies from single cells in colony formation assays. No difference in the overall number of established colonies was observed between 786‐O pBABE‐mock and 786‐O pBABE‐TCF21 cells (*P* > 0.5). However, individual colonies of 786‐O pBABE‐mock cells were larger compared with *TCF21*‐expressing cells (Fig. 2B,C, *P* < 0.01). These data are partly consistent with the proliferation data and support the idea that while *TCF21* overexpression does not seemingly affect cell survival (colony number), proliferation (colony size) is suppressed.

### 3.3 *Vhl*−/− zebrafish pronephros phenotype is not rescued by TCF21

786‐O cells are derived from a ccRCC which, like most ccRCCs, harbors biallelic somatic mutations of the von Hippel‐Lindau (*VHL*) tumor suppressor gene (Gnarra *et al*., 1994). We have previously generated a zebrafish model with biallelic constitutive *vhl* mutations and have reported the embryonic renal phenotype as exhibiting an alveolar hyperplastic morphology (van Rooijen *et al*., 2011). Not much is known about the endogenous *TCF21* expression in zebrafish although lineage tracing experiments suggest a role for TCF21 in epicardial cell fate (Kikuchi *et al*., 2011). Given the apparent effect of *TCF21* on 786‐O *vhl*−/− cells, we questioned whether injection of *TCF21* RNA into our *vhl*−/− zebrafish embryos would partially or completely rescue the pronephric phenotype. To that end, 17 *vhl*−/− zebrafish embryos were injected with human *TCF21* RNA (*n* = 8), or mock‐injected with dye only as a control (*n* = 9) within an hour after fertilization, and imaged 4 days later, when the pronephros phenotype in *vhl*−/− is very clear, with z‐stacks on a confocal microscope. All embryos were genotyped for confirmation after the experiment and only images of confirmed *vhl*−/− were analyzed. No differences were observed in pronephros size between mock‐injected or *TCF21* mRNA‐injected zebrafish embryos (Fig. S2).

### 3.4 *TCF21* expression suppresses migration

**Figure 3:**
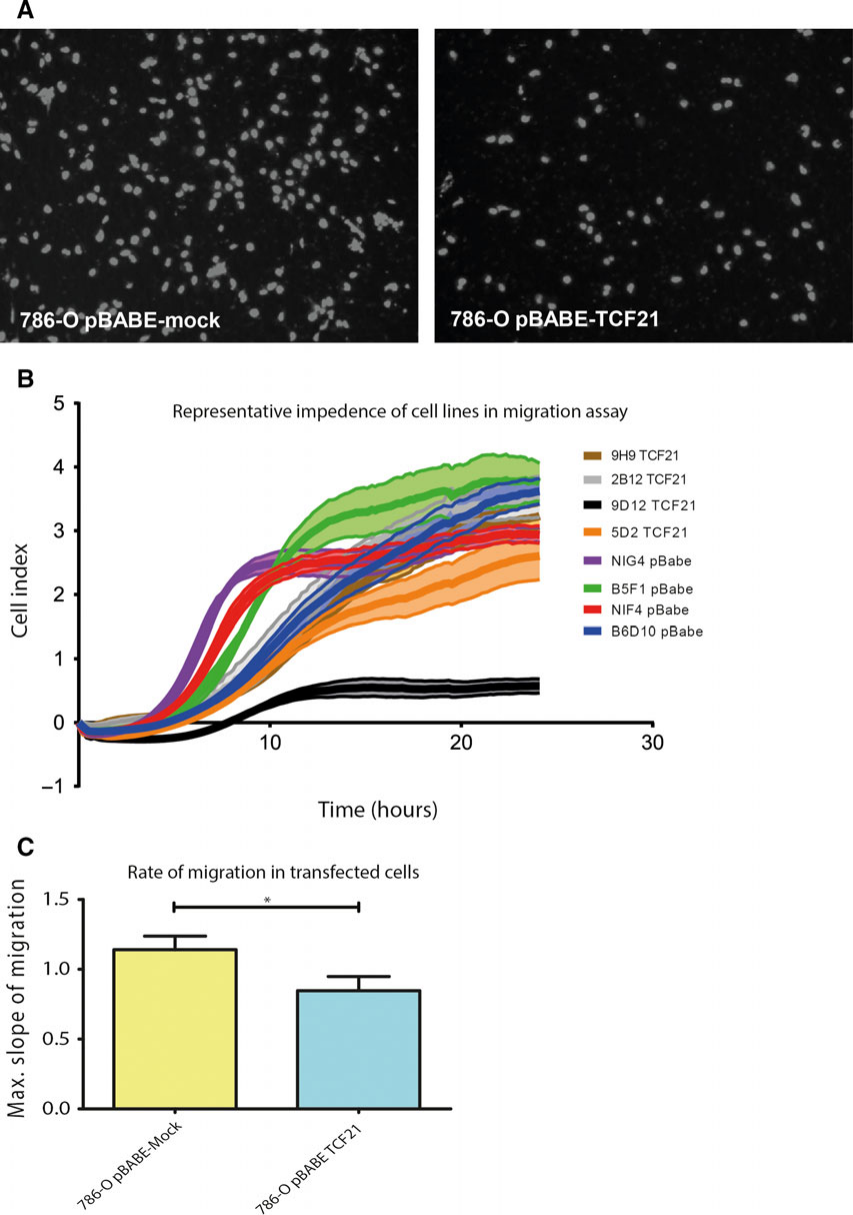
*TCF21* expression reduces migration of cells *in vitro*. (A) Representative photographs of DAPI‐stained nuclei after migration through a Boyden chamber of 786‐O pBABE‐mock cells and 786‐O pBABE‐TCF21 cells. (B) Sample curve of impedance (cell index) from a representative experiment with all clones over 24 h, from which slopes were calculated. (C) The rate of change in impedance of cells across a transwell membrane in an XCeLLigence system indicates that clones expressing *TCF21* migrate significantly slower than pBABE‐mock‐transfected cells. Data are presented as mean ± SEM. *N* = 3 independent experiments; each experiment had four technical replicates. *P < 0.05.

Because the effect of the colony formation assays could only be partially explained by the modest cell proliferation differences observed in Fig. 2A, we hypothesized that the colony area differences (Fig. 2C) might as well be attributed to altered cell migration. Initial analysis of migration using the chemotaxis‐based Boyden chamber assay on two pBABE‐mock clones (N1G4 and B6D10) and two pBABE‐TCF21 clones (5D2 and 9D12) showed a trend toward inhibition of migration in *TCF21*‐expressing cells (analysis repeated for N1G4 and 5D2 clones). Fifty‐nine percent fewer of the *TCF21*‐expressing cells migrated on average (Fig. 3A). To verify these findings, we subsequently serum‐starved all four pBABE‐mock and four pBABE‐TCF21 clones for 24 h and then exposed them to either empty medium or 10% FBS for each clone in triplicate and measured impedance changes 12 times per hour for 24 h using an xCELLigence system. We observed a decrease in cell migration as represented by impedance measurements in the isogenic clones expressing *TCF21* (*P = *0.0495, Fig. 3B,C). Taken together, these data indicate that ccRCC cells become less likely to migrate upon reconstitution of *TCF21* expression.

### 3.5 E‐cadherin is upregulated but mislocalized upon *TCF21* expression

**Figure 4:**
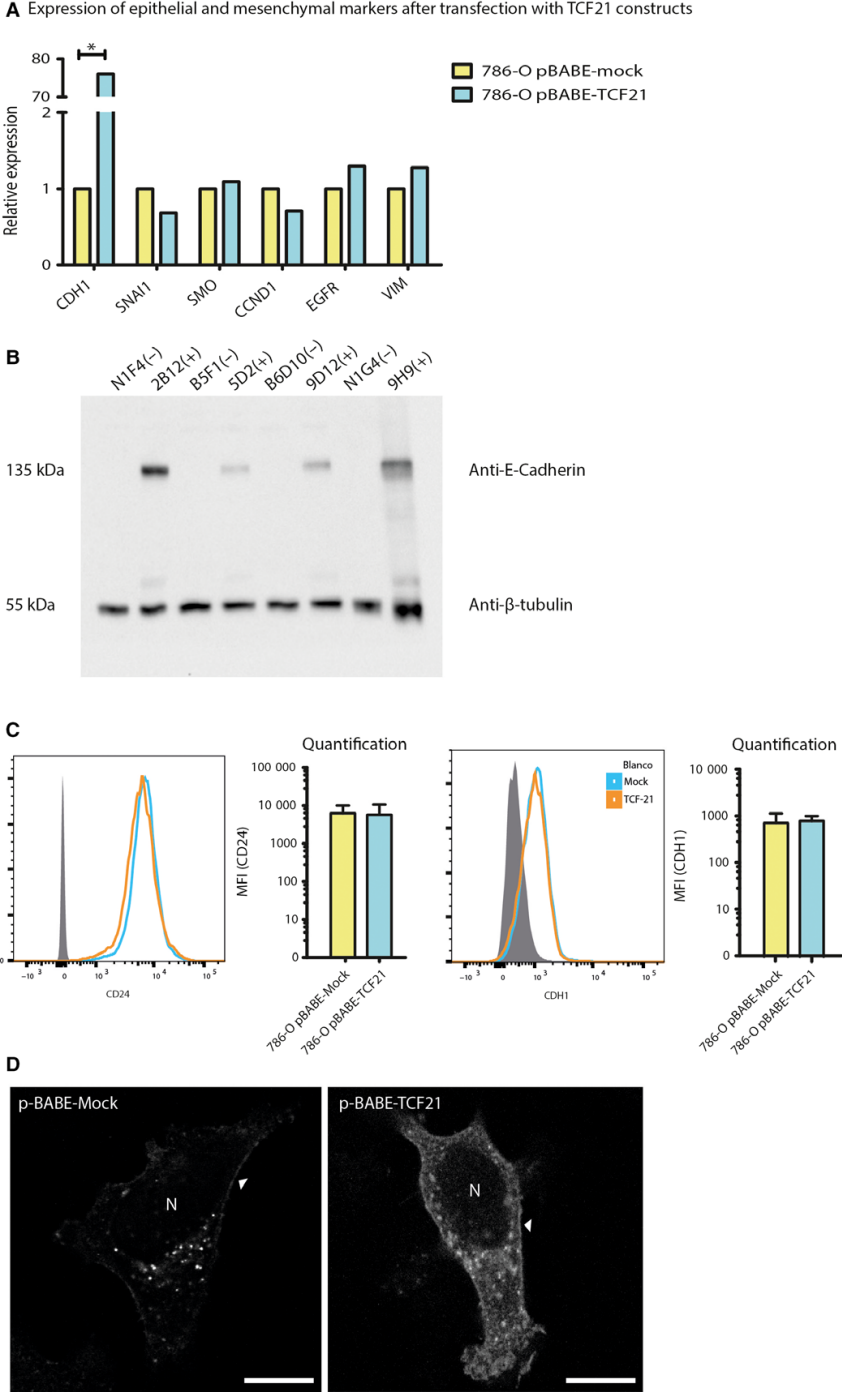
*TCF21* expression and regulation of selected epithelial or mesenchymal markers. (A) mRNA expression levels of E‐cadherin (*CDH1*), *SNAI1*,*SMO*, cyclin D1 (*CCND1*), *EGFR*, and *VIM* in 786‐O pBABE‐mock and 786‐O pBABE‐TCF21 cells, as measured by qPCR. *N* = 2 independent experiments; each performed in triplicate. (B) Western blot of E‐cadherin and ß‐tubulin (loading control) of 786‐0 clones expressing either TCF21 ‘(+)’ or empty vector ‘(−)’. *N* = 2 independent experiments. (C) FACS analyses of the eight isogenic clones indicate no difference in the functional expression of E‐cadherin (*CDH1*) or glycoprotein CD24 in 786‐O pBABE‐mock cells versus 786‐O pBABE‐TCF21 cells. The gray histograms are unstained controls. Next to the histograms are the quantifications of the mean fluorescence intensity (MFI) for the four clones with pBABE‐TCF21 versus pBABE‐mock. Data are presented as mean ± 95% confidence interval. No significant differences were observed. (D) Confocal maximum projection of E‐cadherin (white) in representative 786‐0 pBABE‐mock (clone B6D10) and pBABE‐TCF21 (clone 9H9) cells, showing minimal changes in expression at the membrane (arrowhead) despite global upregulation in cells upregulated in cells expressing TCF21. 10 cells imaged per clone. N = nucleus, *P < 0.05, scale bar = 10 μm.

Epithelial‐to‐mesenchymal transition (EMT) is a reversible process by which fully differentiated cells lose their epithelial features and acquire a migratory mesenchymal phenotype, with a concomitant increased expression of mesenchymal‐associated proteins and decreased expression of epithelial markers. EMT is known to contribute to the metastasis of RCC (Mikami *et al*., 2014), although the underlying cellular and molecular mechanisms have not been clarified yet. The inhibition of migration in ccRCC cells by re‐expression of *TCF21* (Fig. 3) suggests that *TCF21* might function in the renal epithelium by consolidating epithelial characteristics, or alternatively, in repressing mesenchymal characteristics. We investigated mesenchymal and epithelial marker expression in the eight 786‐O clones expressing pBABE‐mock versus pBABE‐TCF21 by qPCR. No changes were observed in mRNA levels of the mesenchymal markers *VIM* and snail (*SNAI1*), but we did observe significant upregulation of epithelial marker E‐cadherin (*CDH1*) both at the mRNA and at the protein level in the clones expressing ectopic *TCF21* (Fig. 4A,B). These data could suggest that cells with hypermethylated *TCF21* promoters are more predisposed to EMT, thereby potentially contributing to the tumor suppressor function of *TCF21* in renal tumors.

In a melanoma cell line (C8161), re‐expression of *TCF21* was described to activate expression of the metastatic suppressor *KISS1* and consequently inhibit motility of the cells (Arab *et al*., 2011; Zhang *et al*., 2012). We investigated *KISS1* expression levels in 786‐O clones expressing pBABE‐mock and clones expressing pBABE‐TCF21; expression levels were undetectably low in both untransfected and transfected 786‐O cells, while being robustly expressed in control human placenta (average *C*_q_ value 21.4).

As consistent upregulation of genes involved in the Sonic hedgehog signaling pathway and Akt cell proliferation pathway has been reported in both CCSKs and ccRCCs (Cutcliffe *et al*., 2005; Dormoy *et al*., 2009; Guo *et al*., 2015), we investigated expression of a few genes involved in these pathways [epidermal growth factor receptor (*EGFR*)*,* smoothened (*SMO*)*, CCND1*] in pBABE‐mock and pBABE‐TCF21 cells; no significant difference in expression was observed (Fig. 4A).

To test the functional relevance of increased *CDH1* expression, we performed fluorescence‐activated cell sorting (FACS) of extracellular epithelial differentiation markers CDH1 (E‐cadherin) and CD24. Each 786‐O clone expressing either pBABE‐mock or pBABE‐TCF21 was stained by directly conjugated fluorescence antibodies for the endogenous extracellular epitope of CDH1 or CD24 and sorted on the strength of the fluorescent signal and side scatter. In contrast to the qPCR data, we observed no differential CDH1 or CD24 protein expression in the pBABE‐TCF21 clones compared to the pBABE‐mock clones (Fig. 4C).

**Figure 5:**
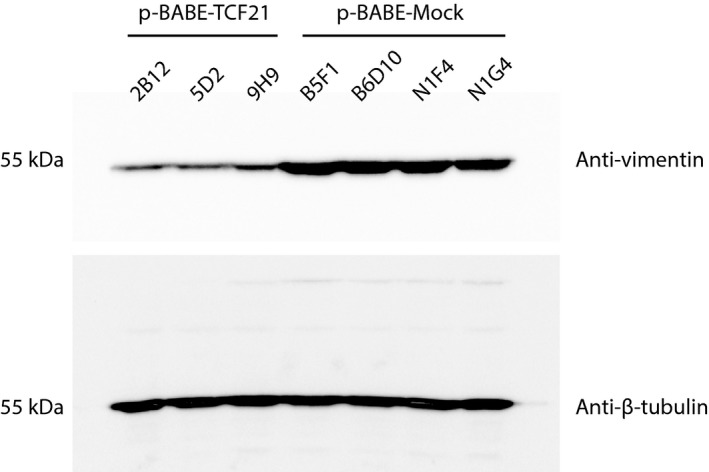
Mesenchymal marker *VIM* is downregulated by TCF21. Three clones expressing pBABE‐TCF21 show reduced levels of *VIM* on western blot as compared to four pBABE‐mock clones. Clone 9D12 was not included in the analysis as it did not express TCF21 at the time lysates were prepared (Fig. S1B). *N* = 2 independent experiments.

Because the upregulation of CDH1 did not correlate with increased CDH1 extracellular epitope, we investigated the subcellular localization of E‐cadherin using immunofluorescence. We observed that although upregulation of E‐cadherin is also observed in 786‐O clones expressing ectopic TCF21, it is not localized at the plasma membrane in correspondingly high levels in both low‐density and confluent cultures (Fig. 4D). We conclude that TCF21 directly affects expression of *CDH1*, although possibly the cancer cell line we used does not have the intrinsic ability to incorporate significantly more E‐cadherin into the plasma membrane. Lastly, we examined levels of mesenchymal marker *VIM* by western blot and observed that 786‐O clones expressing TCF21 (Fig. S1B) display lower levels of *VIM* (Fig. 5).

## 4 Discussion

In the present study, functional experiments demonstrate that reconstitution of *TCF21* expression in 786‐O cells results in decreased clonogenic proliferation and suppressed migration *in vitro*. These data support a tumor suppressor function for *TCF21* in the context of ccRCC, in accordance with several studies that have demonstrated that *TCF21* acts as a tumor suppressor gene in different tumor types. For example, re‐expression of *TCF21* reduced cell growth and colony formation in lung cancer cells *in vitro* and *in vivo* (Smith *et al*., 2006), reduced cell proliferation, promoted apoptosis and suppressed cell invasion and migration in colorectal cancer *in vitro* (Dai *et al*., 2016), and reduced cell proliferation and epithelial–mesenchymal transition in breast cancer cells *in vitro* (Wang *et al*., 2015). In addition, downregulation of *TCF21* through hypermethylation has been reported to be associated with poor outcome in patients with ccRCC and metastatic melanoma (Ye *et al*., 2012).

As reported before, and confirmed in the current study, expression of *TCF21* is (at least partly) repressed by methylation in 786‐O cells (Ye *et al*., 2012). *TCF21* hypermethylation has also been demonstrated in other ccRCC cell lines and human ccRCC tissue samples (Costa *et al*., 2011; Xin *et al*., 2016; Ye *et al*., 2012). Likewise, in other human cancer types, such as gastric cancer, colorectal cancer, melanoma, head and neck cancer, and non‐small‐cell lung carcinoma, hypermethylation is described to be the predominant mechanism for *TCF21* downregulation (Arab *et al*., 2011; Dai *et al*., 2016; Smith *et al*., 2006; Yang *et al*., 2015).

To increase metastatic and invasive capacities, cancer cells often show loss of epithelial and gain of mesenchymal characteristics, which permit invasion along the basement membrane, establishing an opportunity for metastasis (Birchmeier *et al*., 1996; Thiery, 2002). *TCF21* is known to be involved in mesenchymal‐to‐epithelial transition (MET) (Acharya *et al*., 2012; Quaggin *et al*., 1999; Smith *et al*., 2006; Wang *et al*., 2015). Although we did not detect decreased mRNA expression of mesenchymal markers in our 786‐O cells transfected with *TCF21*, our data support a positive association of *TCF21* expression and increased mRNA and protein expression of the epithelial marker E‐cadherin (*CDH1*), which was markedly upregulated in *TCF21*‐expressing 786‐O cells. However, immunofluorescence and FACS experiments demonstrate that despite global upregulation, expression E‐cadherin is not increased at the plasma membrane, which may be cell specific for 786‐O cells. Inactivation of *CDH1* is described to be prominently associated with tumor invasiveness, metastatic dissemination, and poor patient prognosis; the significance of *CDH1* expression for metastatic potential has been shown in a variety of *in vitro* and *in vivo* models (Frixen *et al*., 1991; Onder *et al*., 2008; von Burstin *et al*., 2009). Therefore, increase in *CDH1* expression might be a possible reason for decreased migration of *TCF21*‐expressing 786‐O cells in the current study, through alternative downstream transcriptional pathways, because we did not detect changes in the extracellular epitope for CDH1 (Canel *et al*., 2013; Onder *et al*., 2008). Furthermore, our results that overexpression of TCF21 inhibits migration are in accordance with *in vitro* studies in colorectal cancer cell lines (Dai *et al*., 2016, 2017) as well as siRNA suppression of endogenous TCF21 in renal progenitor cells which increased migration (Plotkin and Mudunuri, 2008). Although re‐expression of *TCF21* in melanoma cells and Caki‐1 ccRCC cells has been described to activate expression of the metastatic suppressor *KISS1* (Arab *et al*., 2011; Zhang *et al*., 2012), in the current study *KISS1* expression does not seem to be involved in the suppressed migration of 786‐O cells transfected with *TCF21*. It would also be of value to explore several recently identified TCF21 targets expressed in renal tubules such as IL6R, SH2B3, and SMG6 and interrogate their contribution to phenotypic differences (Sazonova *et al*., 2015). Further studies are warranted to consolidate these data.

Recently, we reported that CCSKs bearing the *BCOR* internal tandem duplication showed hypermethylation of *TCF21*, while CCSKs bearing the translocation t(10;17)(q22;p13) showed significantly lower methylation levels of the *TCF21*, suggesting that internal tandem duplication of *BCOR* may be directly or indirectly responsible for *TCF21* hypermethylation in CCSKs (Gooskens *et al*., 2016). To functionally validate the tumorigenic role of *TCF21* hypermethylation in CCSKs, CCSK models urgently need to be developed. We explored the possibility of using a *vhl*−/− zebrafish model for reconstitution, but did not observe any phenotypic modulation. Due to the rarity of CCSK and limited availability of histologically verified fresh tissue, it is a challenge to develop such research tools as cell lines or other CCSK models. If we are able to develop efficient and physiologically relevant models through additional studies and could generate similar results in CCSK cells as in ccRCC cells, then treatment with demethylating agents might be an option for these patients. Currently, the most studied and promising demethylating agents are the DNA methyltransferase inhibitors decitabine and azacitidine (Kulis and Esteller, 2010).

## 5 Conclusions

In summary, our study revealed that restoration of *TCF21* expression in 786‐O ccRCC cells results in decreased clonogenic proliferation and migration. CCSK models need to be developed to fully determine the influence of reconstitution of *TCF21* expression in CCSK cells.

## Author contributions

SLG, RP, EJP, RHG, and MMvdHE conceived and designed the project. SLG, TDK, HG, IL, and RHG acquired the data. SLG, TDK, and RHG analyzed and interpreted the data. SG, TDK, RHG, and MMvdHE wrote the manuscript.

## Supporting information


**Fig S1**. (A) Western blot of anti‐TCF21 (18 kDa) on HEK293T cell lysate (far left, positive control for endogenous protein), 786‐0 parental cells (far right, negative control), and clones stably transfected with either empty pBABE‐puro plasmid ‘(−)’, or TCF21 ‘(+)’ in pBABE‐puro. Equal loading is indicated by β‐tubulin staining (55 kDa). Experiment was performed twice independently. (B) Western blot of anti‐TCF21 (18 kDa) with the same lysates used Fig. 5 shows that TCF21 was no longer stably expressed in clone 9D12, which was consequently not loaded on to the blot for *VIM* expression (Fig. 5).

**Fig. S2**. Confocal images of the trunk of *vhl*−/− zebrafish embryos (5 days postfertilization, anterior is to the left in all images) in a background with green fluorescent pronephros (Tg‐*cdh17*:GFP).

**Table S1**. Primer sequences used for qPCR experiments.
