# Supplementary figures and images for: TCF21 hypermethylation regulates renal tumor cell clonogenic proliferation and migration
Source: Mol Oncol. 2017 Dec 14;12(2):166–79. doi: 10.1002/1878-0261.12149 (PMC5792742; doi:10.1002/1878-0261.12149)

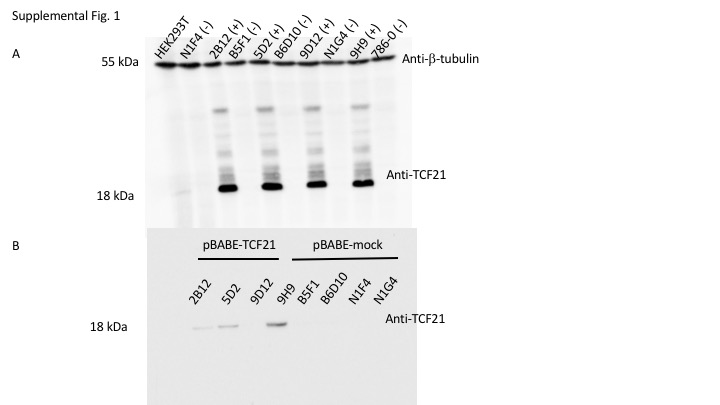

Supplement: Supplementary file 1 — Fig S1. (A) Western blot of anti‐TCF21 (18 kDa) on HEK293T cell lysate (far left, positive control for endogenous protein), 786‐0 parental cells (far right, negative control), and clones stably transfected with either empty pBABE‐puro plasmid ‘(−)’, or TCF21 ‘(+)’ in pBABE‐puro. Equal loading is indicated by β‐tubulin staining (55 kDa). Experiment was performed twice independently. (B) Western blot of anti‐TCF21 (18 kDa) with the same lysates used Fig. 5 shows that TCF21 was no longer stably expressed in clone 9D12, which was consequently not loaded on to the blot for VIM expression (Fig. 5). [file MOL2-12-166-s001.jpg]

Supplemental figure 1

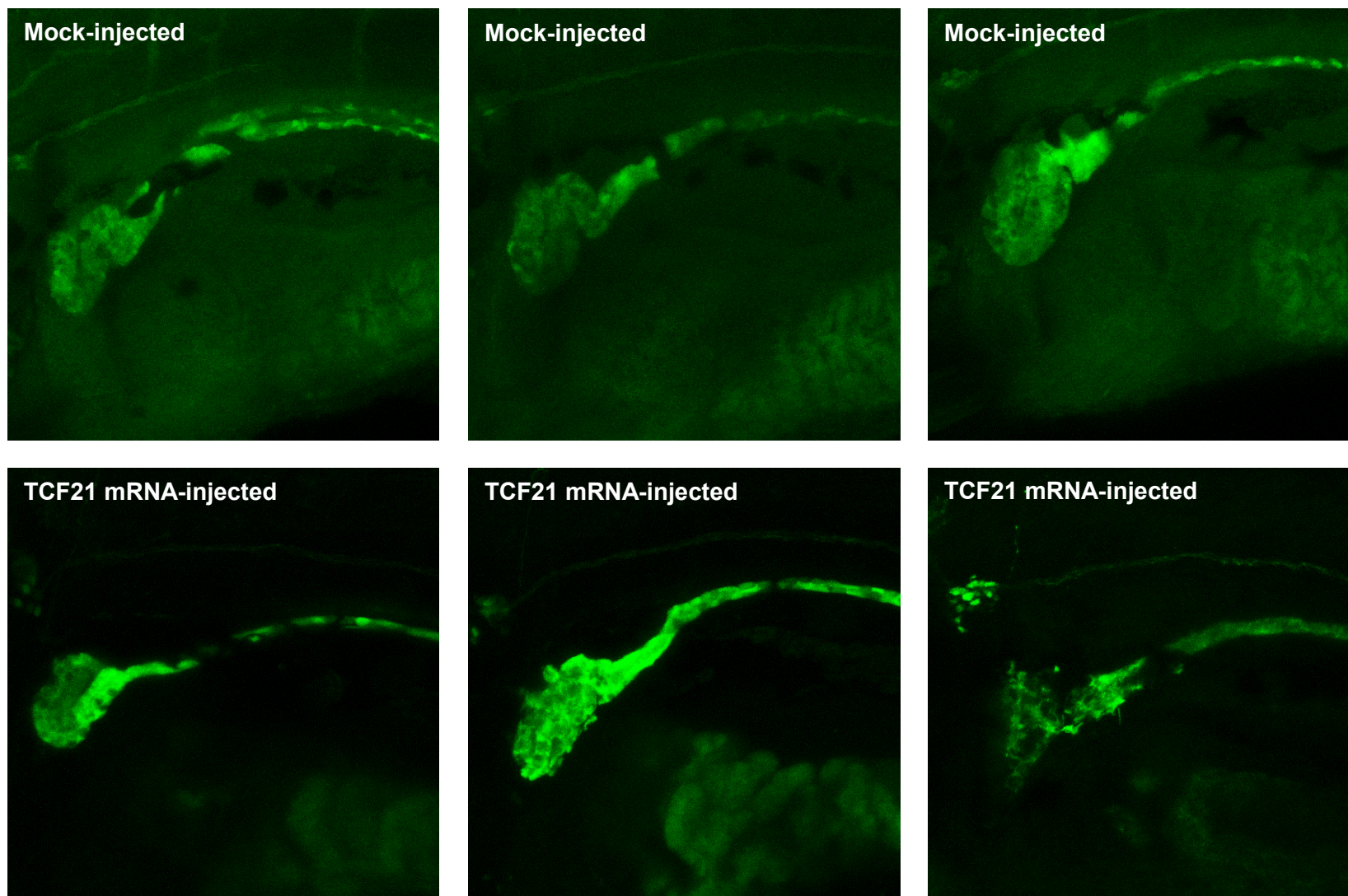

Supplement: Supplementary file 2 — Fig. S2. Confocal images of the trunk of vhl−/− zebrafish embryos (5 days postfertilization, anterior is to the left in all images) in a background with green fluorescent pronephros (Tg‐cdh17:GFP). [file MOL2-12-166-s002.pdf]
