# Supplementary material for: TCF21 hypermethylation regulates renal tumor cell clonogenic proliferation and migration
Source: Mol Oncol. 2017 Dec 14;12(2):166–79. doi: 10.1002/1878-0261.12149 (PMC5792742; doi:10.1002/1878-0261.12149)
Supplement: Supplementary file 3 — Table S1. Primer sequences used for qPCR experiments. [file MOL2-12-166-s003.pdf]

Supplemental Table 1

Gene	Forward primer	Reverse primer
GAPDH	GGAGCGAGATCCCTCCAAAAT	GGCTGTTGTCATACTTCTCATGG
TCF21	TCCTGGCTAACGACAAATACGA	TTTCCCGGCCACCATAAAGG
EGFR	TTGCCGCAAAGTGTGTAACG	GTCACCCCTAAATGCCACCG
SMO	GAAGTGCCCTTGGTTCGGA	GCAGGGTAGCGATTCGAGTT
CDH1	CGAGAGCTACACGTTCACGG	GGGTGTCGAGGGAAAAATAGG
VIM	AGTCCACTGAGTACCGGAGAC	CATTTCACGCATCTGGCGTTC
CCND1	GATCAAGTGTGACCCGGACT	TCCTCCTCTTCCTCCTCCTC
SNAI1	ACCCACACTGGCGAGAAG	ATTCCATGGCAGTGAGAAGG
KISS1	ACCTGCCTCTTCTCACCAAG	TAGCAGCTGGCTTCCTCTC
